# Thirty-Five Years of Gossypiboma

**DOI:** 10.7759/cureus.94478

**Published:** 2025-10-13

**Authors:** Roberto Passa, Michela Angelucci, Chiara Pagnoni, Sergio Valeri

**Affiliations:** 1 General Surgery, Fondazione Policlinico Universitario Campus Bio-Medico, Rome, ITA; 2 General Surgery, Università Campus Bio-Medico di Roma, Rome, ITA

**Keywords:** abdominal surgery, general surgery, gossypiboma, retained surgical item, textile matrix

## Abstract

The term gossypiboma refers to a textile matrix object left in a body cavity during surgery. It is often mistaken for other pathologies and symptoms can be very heterogeneous. Retained surgical items often have legal as well as clinical consequences. To reduce the incidence of gossypiboma, safety procedures have been proposed that the surgical team must implement. In case of an occasional finding during another procedure, it is necessary to share the most correct management. The case we present involves a patient in whom an intra-abdominal mass was identified during a laparoscopic cholecystectomy. The initial decision was to perform a biopsy of the mass and postpone the definitive surgery. Histological examination provided the diagnosis of gossypiboma. The patient’s medical history revealed that the surgical item had been retained for 35 years. Subsequently, we performed a second operation in which the gossypiboma was removed at the same time as the cholecystectomy.

## Introduction

A retained surgical item (RSI) is the term used when an object is accidentally left inside a patient's body during a surgical procedure [1]. The incidence varies between 1/100 and 1/1500 laparotomies [2]. The morbidity of this condition varies between 11% and 35% and depends on the composition of the foreign body and the duration of its retention [3,4]. The differential diagnosis can be very challenging and is often made postoperative. Nowadays, we know that RSI is more closely related to the culture of the surgical team than to the characteristics of the patient [5]. The average cost of removing an RSI is estimated to be $63,631, not including the high legal costs that often follow [6]. When the retained object has a cotton matrix and generates a surrounding inflammatory reaction, it is defined as a "gossypiboma" (from Latin “gossypium” meaning “cotton” and Swahili “boma” meaning “place of concealment”) [7]. Despite the safety systems proposed over the years, RSI remains an incompletely solved problem. The aim of this report is to illustrate the appropriate management of a mass of indeterminate nature that may be encountered during surgical procedures. To the best of our knowledge, the gossypiboma presented here is one of the RSI discovered after the longest postoperative interval reported so far.

## Case presentation

A 45-year-old male patient presented to our department with symptomatic cholelithiasis. An abdominal ultrasound revealed two gallbladder stones measuring 16 and 20 mm. No other abdominal findings were reported. No palpable masses were detected on physical examination. The patient's medical history included previous open appendectomy performed 35 years earlier. Preoperative parameters are shown in Table 1.

**Table 1:** Lab investigations Laboratory findings of the patient. No alterations in inflammation indices, liver and pancreatic tests were found

Parameter	Result	Normal range
C-reactive protein	0.1 mg/dL	<0.5 mg/dL
Complete blood count		
Hemoglobin (HGB)	13.5 g/dl	13.5-17.5 g/dl
Red blood cell (RBC)	4.51x10^6^/uL	4.3-5.5x10^6^/uL
White blood cell (WBC)	7.07x10^3^/uL	4-10x10^3^/uL
Hematocrit (HCT)	38.8%	40-50%
Mean corpuscular volume (MCV)	86 fL	83-101 fL
Neutrophils (%Neut)	73.9%	40-80%
Lymphocytes (%Lymp)	9.98%	20-40%
Monocytes (%Mono)	9.88%	2-10%
Eosinophil (%Eos)	5.63%	1-6%
Basophil (%Bas)	0.65%	0.3-1%
Liver function test		
Alanine transaminase (ALT)	43 U/L	0-55 U/L
Aspartate transaminase (AST)	31 U/L	5-34 U/L
Total bilirubin	1.1 mg/dL	0.3-1.2 mg/dL
Direct bilirubin	0.4 mg/dL	0-0.5 mg/dL
Alkaline phosphatase (ALP)	55 U/L	53-128 U/L
Pancreatic function test		
Amylase	43 U/L	25-125 U/L
Lipase	33.91 U/L	0-59 U/L
Renal function test		
Creatinine	0.84 mg/dL	0.73-1.18 mg/dL
Blood urea nitrogen	17 mg/dL	19-43 mg/dL
Serum electrolytes		
Sodium	137 mmol/L	136-145 mmol/L
Potassium	3.8 mmol/L	3.5-5.1 mmol/L
Chloride	103 mmol/L	98-107 mmol/L

The patient’s body mass index (BMI) was 23.6 kg/m^2^. There were no contraindications to cholecystectomy. During the early laparoscopy, a solid, round mass measuring 70 mm in diameter and of uncertain nature was found in the right iliac fossa, strongly adherent to the small bowel. We decided not to proceed with cholecystectomy and a laparoscopic biopsy was performed using Tru-Cut needle (Bard® Max-Core® 18 g x 25 cm. Ref: NC1825; Bard, Murray Hill, New Providence, NJ, USA).

**Figure 1:**
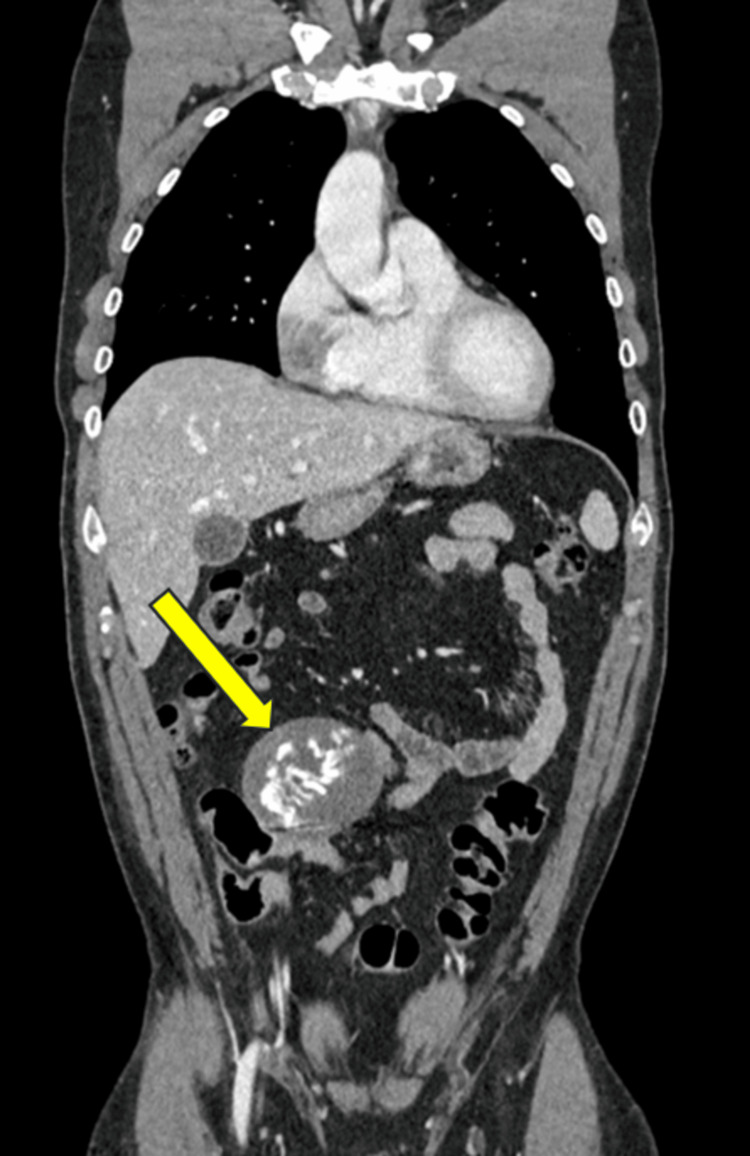
CT scan shows the gossypiboma in the right iliac fossa in coronal view. The yellow arrow indicates the gossypiboma.

**Figure 2:**
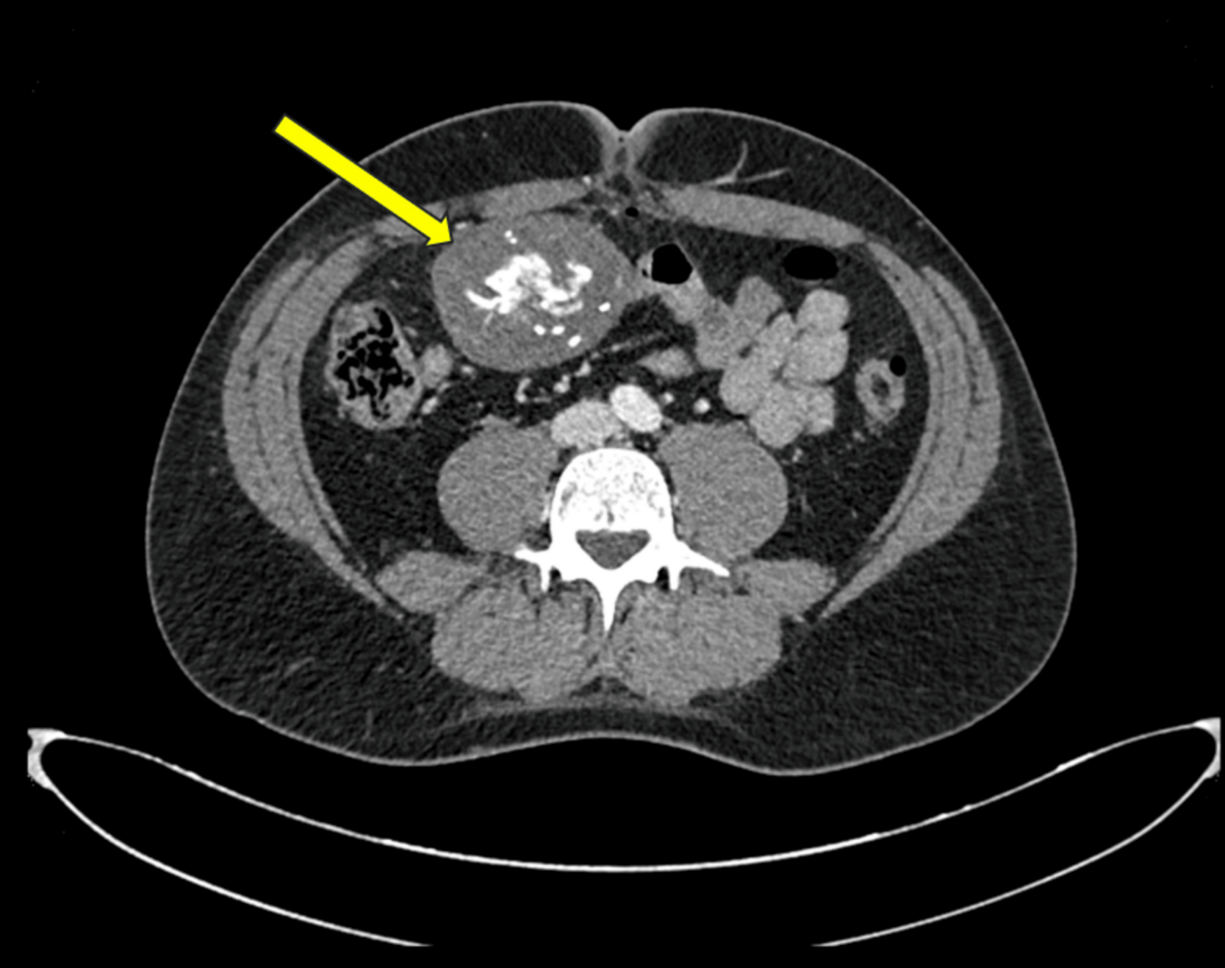
CT scan shows the gossypiboma in the right iliac fossa in axial view. The yellow arrow indicates the gossypiboma.

A computed tomography (CT) scan confirmed a solid, hypodense mass with multiple calcifications, measuring 70×60 mm (Figures 1, 2). No other suspicious findings were reported. The patient was discharged two days later in good clinical condition. The histological result revealed the presence of acellular, amorphous, and partially calcified material encapsulating fragments of fibrous, birefringent material of likely exogenous origin. In consultation with the pathologists, the biopsy sample was deemed representative due to the presence of textile material and the surrounding granulomatous reaction, which allowed for a definitive diagnosis. The quality of the specimen was therefore considered adequate. The nature of the mass was explained to the patient. He decided to remove it together with the gallbladder.

**Figure 3:**
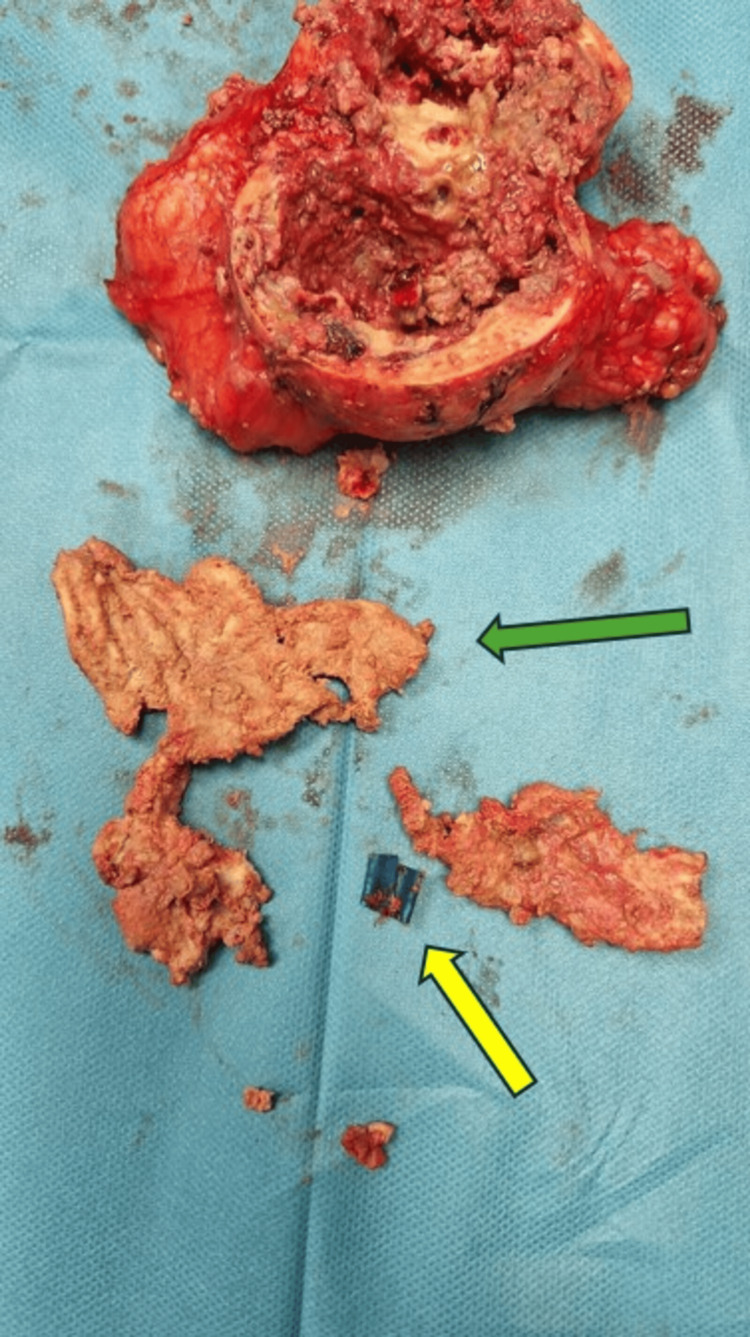
A macroscopic evaluation of the histological sample shows caseous necrosis within the mass caused by textile (green arrow) and plastic material (yellow arrow)

**Figure 4:**
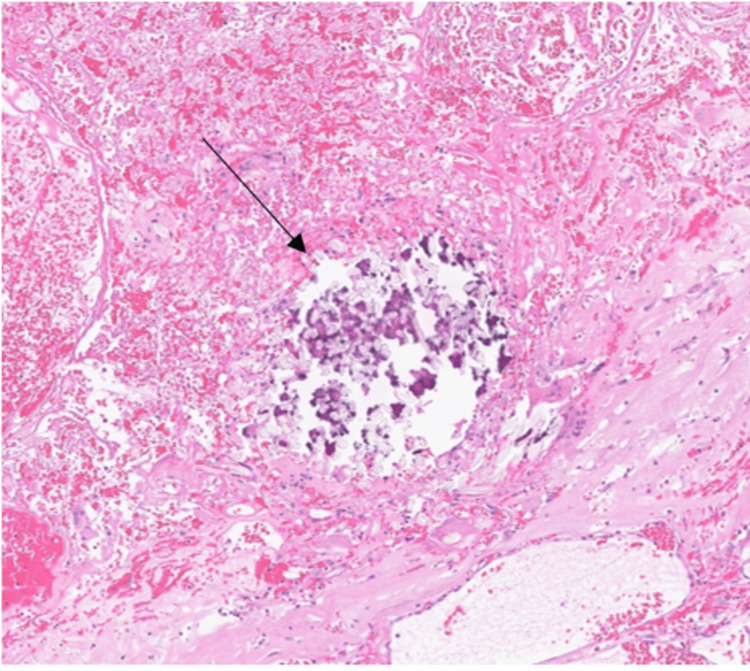
Hematoxylin-eosin (H&E) stain showing a foreign-body granulomatous reaction (black arrow) characterized by multinucleated giant cells and dystrophic calcification encasing residual surgical stitches (20x magnification)

The second surgery was carried out two months later. First, we performed a laparoscopic cholecystectomy using the French technique. Then, a supra-umbilical laparotomy was necessary to remove the mass. A thorough viscerolysis was performed. The operating time was 210 minutes, and no significant blood loss occurred. A material resembling surgical gauze mixed with plastic elements was found within the mass, which had caused caseous necrosis (Figure 3). The final histological examination confirmed the diagnosis of a foreign body granuloma (Figure 4). The postoperative course was uneventful. The patient was discharged six days later.

## Discussion

Gossypiboma refers to a mass within the human body composed of a textile matrix and surrounded by an inflammatory granuloma [8]. The first documented case of RSI was published by Wilson in 1884 and documented a gauze left after a laparotomy [9]. The incidence of this condition is not well understood, but it is influenced by morbid obesity and complexity of the surgical procedure. It appears to be both patient-specific and procedure-specific [10-11]. However, some studies have highlighted contrasting findings [12].

Furthermore, the advent of minimally invasive surgery has not reduced the incidence of RSI [13]. The material retained in the body first generates an exudative reaction. Subsequently, a fibrinous reaction occurs, forming a solid capsule around the foreign tissue [14]. The clinical presentation is highly varied. The onset of symptoms is subjective and can occur either after a few days or many years. This condition rarely leads to immediate and recognizable complications. Often, its manifestations are subclinical and generally related to the granulomatous reaction. As a result, symptoms tend to be nonspecific, such as a painless palpable mass, intestinal subocclusion or perforation, or wound dehiscence. Sometimes, gossypiboma can mimic benign or malignant lesions as documented by Manzella et al. [15]. A detailed patient history may help the surgeon consider this possibility and direct the diagnostic workup toward potential underlying causes. Often, the diagnosis is incidental.

Our case confirms that the diagnosis is often incidental despite many years of gauze retention. Evidence from literature highlights that this condition is frequently preventable with appropriate security measures. Several solutions have been proposed to reduce the incidence of RSI. One of these is counting the surgical sponges before and after the procedure. If the count does not match, the entire surgical team is responsible for finding it [16]. Another preventive measure involves the use of radiopaque threads in surgical sponges, allowing for the identification of retained items through intraoperative imaging. However, this procedure is secondary to the proper counting of surgical materials. Since it is still a widespread problem today, it is necessary to understand whether RSI should be considered a possible complication during surgery or an act of gross medical negligence. Most legal rulings lean towards negligence. In addition to the morbidity and mortality concerns for the patient, gossypiboma can also lead to allegations of surgeon malpractice [17].

## Conclusions

RSI remain a major issue in surgical practice worldwide despite the implementation of modern safety protocols. This case highlights how symptoms may be entirely absent even after decades and it illustrates the appropriate management when an unexpected mass is encountered intraoperatively. To the best of our knowledge, this represents one of the longest documented cases of gossypiboma retained in a body cavity.
